# Physicians’ perceptions of the implementation of the serious illness care program: a qualitative study

**DOI:** 10.1186/s12913-023-10419-5

**Published:** 2023-12-12

**Authors:** Susanna Pusa, Rebecca Baxter, Anna Sandgren

**Affiliations:** Center for Collaborative Palliative Care, Department of Health and Caring Sciences, Linnaeus University, Växjö, Sweden

**Keywords:** Advance care planning, Health communication, Implementation science, Palliative care, Physicians, Serious Illness care program, Serious Illness conversations

## Abstract

**Background:**

Conversations about goals, values and priorities with patients that are seriously ill are associated with improved palliative healthcare. The Serious Illness Care Program is a multi-component program that can facilitate more, better, and earlier conversations between clinicians and seriously ill patients. For successful and sustainable implementation of the Serious Illness Care Program, it is important to consider how stakeholders perceive it. The aim of our study was to explore physicians’ perceptions and experiences of implementing the Serious Illness Care Program.

**Methods:**

Data were collected through four focus group discussions with physicians (n = 14) working at a hospital where the Serious Illness Care program was in the process of being implemented. Data were analyzed with inductive thematic analysis.

**Results:**

Physicians’ perceptions of the implementation encompassed three thematic areas: hovering between preparedness and unpreparedness, being impacted and being impactful, and picking pieces or embracing it at all.

**Conclusions:**

This study identified key aspects related to the individual physician, the care team, the impact on the patient, and the organizational support that were perceived to influence the implementation and sustainable integration of the Serious Illness Care Program. Describing these aspects provides insight into how the Serious Illness Care Program is implemented in practice and indicates areas for future training and development.

**Trial registration:**

Not applicable.

**Supplementary Information:**

The online version contains supplementary material available at 10.1186/s12913-023-10419-5.

## Background

Seriously ill patients have a high risk of mortality and are negatively impacted in terms of functioning and quality of life [1]. This patient group and their caregivers are recognized as requiring timely and high-quality care, but it can be difficult to coordinate care when systems are not in place to support integration of care and treatment plans. The Serious Illness Care Program (SICP), developed by Ariadne Labs, aims to prepare clinicians with the knowledge and skills to provide more, better, and earlier serious illness conversations (SIC) [2]. This multi-component program is comprised of clinical tools, clinician training, and systems change [3]. The clinical tools include resources, such as the Serious Illness Conversation Guide (SICG) and the family guide that offers patient-informed language and structured suggestions for discussing goals, values, preferences, and priorities. The training program provides clinicians with information about SIC, practice conducting SIC, and a reference guide that summarizes the training program and offers advice for future conversations. To integrate the SICP into a given healthcare setting, changes must be implemented at systems-level. Such changes include processes for identifying suitable patients for SIC, inviting them to participate, documentation of the SIC in the medical record, and procedures for disseminating information among the wider healthcare team [3].

It is important to evaluate implementation processes in healthcare settings when attempting to improve the feasibility, effectiveness and sustainability of interventions [4]. To improve understanding of implementations and their possible outcomes, process evaluations should be undertaken [5]. Process evaluations are needed to improve the understanding of implementations and their possible outcomes [6]. Exploring key stakeholder perceptions can improve implementation strategies by giving insight into possible barriers and facilitators [6]. Stakeholder evaluations are particularly important in complex multi-component systems-level implementations such as the SICP. Concerted effort is required to successfully implement the SICP in clinical practice as challenges and barriers can arise within patient, clinician, and organizational domains [7]. Exploring physicians’ perceptions and experiences of the implementation of the SICP is essential to informing improved integration strategies across clinician, team, and organization levels. The aim of this study was therefore to explore physicians’ perceptions and experiences of the implementation of the Serious Illness Care Program.

## Methods

### Study design

This qualitative study utilized an inductive design with focus group discussions. Focus group discussions allow participants to interact and discuss a range of topics related to the SICP implementation, resulting in rich and detailed data [8]. Inductive reasoning seeks to generate insights and interpretations about phenomena based on data-driven participant experiences [9, 10]. The COREQ guidelines [11] were used to report the study.

### The Serious Illness Care Program (SICP) implementation

The SICP is a multi-component structured communication-based intervention that aims to facilitate conversations with seriously ill patients in order to improve their care and quality of life [2]. The SICP originates from Ariadne Labs in Boston, USA [3, 12]. The program has been adapted for the Swedish healthcare context, including a translation of the SICG. The SICP was implemented in 20 units at two hospitals in southern Sweden in 2017 and 2018. System-level changes and multi-level collaboration were fostered between researchers, clinical healthcare staff, and hospital administrators.

Physicians attended a one-day (8 h) training in SIC conducted by two facilitators with experience in serious illness communication. One of the facilitators was a palliative care physician and the other facilitator was a behavioral therapist. The training focused on how to carry out SIC and how to respond to patient emotions during the conversation. The training included lectures in SIC theory, conversation role-play with professional actors, and coaching from the facilitators. After the training the physicians met with the facilitators to discuss patient identification criteria to trigger SIC. Eligibility for SIC was indicated if the physician responded *no* to the ‘surprise question’: “Would you be surprised if this patient died within the next year?” [13]. Physicians could also use their own discretion and invite patients who did not meet the surprise question criteria to have a conversation if they felt the patient would benefit from SIC. For instance, patients with critical loss could also be eligible for SIC. After patients were identified, Registered Nurses provided verbal and written information about SIC and an offer to have a conversation with their physician about their goals and priorities. The physicians were encouraged to use the SICG and document the conversation in the medical record using a template.

### Participants

Participants were recruited using purposive sampling. The inclusion criteria were: qualification as a physician and participation in the SICP implementation. After the training in SIC, one of the facilitators contacted the department managers who invited the physicians to take part in a focus group discussion. As this was an open invitation, characteristics of non-participants remain unknown. A total of 14 physicians participated in four focus groups (group 1, n = 5; group 2, n = 4; group 3, n = 3; group 4, n = 2). Reasons for non-participation were not explored. Physicians from focus group 1 worked in the palliative care team, physicians from focus group 2 worked in hematology, physicians from focus group 3 worked in cardiology, and physicians from focus group 4 worked in endocrinology. The participants consisted of 9 men and 5 women whose ages ranged from 39 to 61 (mean, 51 years).

### Data collection

A semi-structured discussion guide was used during the focus group discussions (see Supplementary File A). The discussion guide was comprised of questions connected to perceptions and experiences of the implementation of the SICP, such as the SIC training session, the SICG, identification of eligible patients, and conducting the conversations. The discussions were held between September 2017 and February 2018 by two female researchers, one with a background in nursing and the other in physiotherapy, both of whom had experience in conducting focus group discussions. The moderators had no previous association with the participants prior to the focus group discussion. The focus group discussions lasted between 37 and 61 min, with an average duration of 44 min. The discussions were held in a room at their hospital workplace.

### Data analysis

The focus group discussions were the unit of analysis and were transcribed and then analyzed with thematic analysis. The data were analyzed using a six-phase framework [9]. In the first phase, *familiarizing yourself with the data*, the transcribed discussions were read in an open way to get to know the data and devise initial ideas. During the second phase, *generating initial codes*, segments of the text associated with the same central meaning were condensed and labeled. During the third phase, *searching for themes*, the codes were organized into potential subthemes. In the fourth phase, *reviewing the themes*, the themes and subthemes were examined in relation to the extracts and the text as a whole. Patterns among the codes were identified and the codes were merged in the fifth analysis phase, *defining and naming* themes and subthemes [9]. Throughout the analysis process, and when undertaking the sixth and final phase, *producing the report*, the authors discussed the results in relation to the overall aim, with any discrepancies resolved by consensus (cf. 9).

## Results

Physician perceptions of implementing the Serious Illness Care Program were found to be associated with (a) hovering between preparedness and unpreparedness, (b) being impacted and being impactful, and (c) picking pieces or embracing it at all. For an overview of the themes and subthemes, see Table 1.

**Table 1:** Overview of the themes and subthemes

Themes	Subthemes
*Hovering between preparedness and unpreparedness*	- Uncovering strengths and weaknesses - Having the courage to engage
*Being impacted and being impactful*	- Recognising outcomes as a (de)motivator - Viewing team culture as a possible accelerator - Needing structured support
*Picking pieces or embracing it at all*	- Making adaptations - Considering practical adjustments

### Hovering between preparedness and unpreparedness

Hovering between preparedness and unpreparedness included professional readiness to conduct SIC, recognition and expression of vulnerability, discovering one’s own communication assets, and developing the courage to undertake SIC in practice.

### Uncovering strengths and weaknesses

Physicians described several personal and professional aspects that contributed to an awareness of one’s own strengths and weaknesses in SIC. The SIC training provided an opportunity to discover, acknowledge and develop communication skills. Even though the feeling of being exposed was experienced as uncomfortable at times, moving out of one’s comfort zone and uncovering strengths and weaknesses was described as a natural part of development. Furthermore, altered perspectives emerged through exposing oneself together with an increased understanding of one’s capabilities. Individual communication weaknesses that were identified in the training sessions included aspects related to the SIC approach, SICG questions, interplay and responsiveness between the physician and the patient, and perceived patient needs. In addition to verbal communication, body language, active listening, and following the patient narrative were expressed as important components. Realizing one’s weaknesses meant that these aspects could be mitigated in future SIC.

*So, it’s very good to have this kind of training, because you get to know yourself - and you see your strengths and you see your weaknesses. And you get good tips on how to avoid pitfalls and how to perhaps get out of them…* (focus group 4)

Building awareness around one’s strengths and weaknesses was thought to influence confidence, self-efficacy, and skills development. Physicians also reflected on how the training validated their competence to communicate with patients about their goals and values. Competence came with practice, and the training enhanced preparedness to have SIC. Preparedness was described as individual as well as situational and could depend on motivations connected to one’s values and interests.

*Then it doesn’t always help, right away, that you become aware of your weaknesses. They don’t just disappear all at once. But it helps in any case. It is easier to do something about them if you know about them*. (focus group 4) 

### Having the courage to engage

Having the courage to undertake and engage in SIC in real-world situations following the training was described as an essential part of implementing the SICP. It was important to test oneself by practicing SIC, while also being aware that failure was a possibility. Feelings of nervousness, avoidance, and performance anxiety were identified when taking on the challenge of engaging in SIC. It was necessary to develop courage to try and implement SIC in everyday clinical practice. While physicians expressed that having SIC might not be comfortable, it was important to avoid presenting SIC as something big or difficult. Rather, by recognizing the value of their work for patient care the physicians developed more comfort with the concept of SIC which was expressed as ‘easing the pressure’. This change in mindset enabled physicians to not be afraid of the conversation.

*…how you ask the questions and also then know how to respond to it, not to be afraid of it. I think that’s what I’ve learned. And I also practiced that in the conversation.* (focus group 3)

### Being impacted and being impactful

Being impacted or impactful referred to the reciprocal influence of others and the organization, and vice versa. Including the physician’s perceptions of possible outcomes, team culture impact and the organizational support.

### Recognizing outcomes as a (de)motivator

Physicians recognized that SIC had the power to promote patient empowerment and enable humanizing care. The perceived value of SIC for patients and families also functioned as a motivator to implement SIC, because gaining insight into their wants and needs was viewed as beneficial for everyone. In this way, SIC was viewed in favor of the traditional way of working which did not routinely set aside time to learn patient and family values in relation to their care.

*Bring out the patient’s will, so to speak - what she experienced as the biggest problems, what were her fears and what was she most sad about.* (focus group 3)

However, even though SIC were perceived to be beneficial, physicians also described fear of provoking negative reactions in patients, such as anxiety, worry, and taking away hope for the future. Handling emotions and the topic of prognosis provoked a variety of feelings for physicians and were perceived as being important but challenging. Fear of negative outcomes acted as demotivator for implementing SIC.

### Viewing team culture as a possible accelerator

The influence of culture and subcultures were expressed as having the potential to accelerate and facilitate the SICP implementation, including interpersonal relations, consensus views, and teamwork. The presence and actions of individuals were also seen as influential and motivational for the group as a whole. Team culture was associated with the overall success of the implementation, such as shared value systems of SIC being concordant with their professional beliefs. It was also associated with specific parts of the implementation process, such as training and team-based approach to patient identification with subsequent invitation and information provision about the SIC. It was important for SIC to be collectively viewed as an effective communication strategy so that this attitude could be promoted in their daily work and shared within the team culture. The whole care team was seen having a key function in the implementation of SIC, specifically in the identification of eligible patients, but also in inviting and giving information about the SIC to patients and families.

The influence of the training session was connected to an open culture where expression of insecurity or the need for support was allowed. The learning climate was described as supportive, permissive, and safe. Physicians could feel insecure during the training, but this was mitigated by a feeling of safety within the team. The perceived psychological security in the team, together with the safe learning space, meant that physicians could learn by seeing how others acted during the training session and gaining feedback from them.

*…and you can hear what the others think. So, opinions from colleagues, from the facilitator, from actors - and it’s maybe not every day you get that kind of direct feedback about the conversation...* (focus group 3)

### Needing structured support

Structural characteristics and preparedness of organizational factors and processes were perceived to influence the implementation. Organizational preparedness was described in relation to workflow, where having clear processes was necessary to support the implementation. Conversely, organizational inertia was experienced to hinder the implementation. The traditional workflows were described as somewhat inflexible and slow. Structural characteristics such as high staff turnover, lack of sufficient time, and insufficient information flows were perceived to be barriers to SICP implementation.

Clarity was required regarding how to identify eligible patients efficiently and how to identify the most suitable person to lead the SIC. To increase feasibility continuous structural support was needed, for instance, through ongoing training and opportunities for peer support. Physicians expressed a desire for easily accessible forums where they could discuss and reflect on their experiences with colleagues. To support professional development in SIC competencies, repeated training with gradually increasing difficulty was suggested. This indicates that the training session was seen by some as a starting point for skills development.

Physicians expressed the need for more support in the form of prompting SIC, structured reminders, established implementation goals including short-term milestones, and clear instructions for specific tasks. This could also include an explicit time period to accelerate the implementation. Having someone there to represent the SICP could support physicians to undertake SIC because that person would be present and visible on site to activate and support the physicians.

*You know, I think that if you had received an instruction like this “take the next week and everyone has to come up with five suggestions”, which doesn’t have to be their own patient, “and then we’ll discuss them at the end of the week”. [general agreement]. So, that would probably have kicked off the process quite a bit too* (focus group 2)

### Picking pieces or embracing it all

Adjusting SICP intervention characteristics was described in relation to how it was applied in clinical practice, and the changes that the physicians made to support the adaptations and feasibility of the implementation.

### Making adaptations

Adaptations to the structure and content of SIC varied from picking single questions, to following the SICG systematically, and everything between. Adherence to the full SICP protocol also varied, with physicians describing differences in the ways they prepared the patient, for example by providing information about the content and purpose of the conversation. They also described providing written information that the patient could share with their family members, conducting the conversation using the structured SICG, and documenting the conversation in the medical record.

During the SIC some physicians described having the SICG in front of them, while others simply read it through before the conversation. Likewise, physicians used the SICG questions and responses in alternative ways, for example by asking some SICG questions during their first meeting with a patient, during subsequent meetings, or spontaneously. The SICG questions were experienced to be versatile as they could be used face-to-face or over the telephone. Communication methods and subjects changed over the course of the implementation as the physicians became more familiar with the SICP and adapting SIC. Some conversation aspects identified as being more important, such as understanding the patient’s view and inviting self-reflection about what mattered most for them. Over time, they could adapt their approach and focus more holistically on the patient’s narrative including their emotions, experiences, priorities, and goals.

*…I just wrote down some keywords. So, I didn´t follow the structure to the letter, but I used the phrases because I remembered roughly how they were and how to deal with emotions and how to respond... * (focus group 3)

### Considering practical adjustments

Considering practical adjustments includes possible adaptations that could be made to the SICP to harmonize with the work context, such as processes to suit specific healthcare settings, patient groups, and individual patients. Adjustments were described in relation to different SICP processes, primarily regarding identifying and inviting patients for SIC. Physicians stated that it could be difficult to find the ‘right’ patients for SIC using the recommended “surprise question” because it required them to take a position on whether the patient was likely to die in the next year. Additionally, there was a level of complexity when deciding if the patient was suitable for SIC based on their perceived need, their psychological status, and their illness phase. Physicians felt that such criteria could be too broad or too constrictive.

*Yes, actually, if you let go of the barriers, you could imagine that you could take any patient with a chronic malignant disease who has received one or two lines of treatment and who has that experience and … then you don’t have that one-year question left anymore…* (focus group 2)

Physicians discussed the importance of assessing the patient’s condition because patient stability or instability would impact their eligibility. A ‘stable patient’ was described as having controlled symptoms, no acute deterioration, and no urgent change of treatment plan. So-called stable patients were good candidates for SIC, however, there was some uncertainty about eligibility for patients with acute physical symptoms, such as shortness of breath or unstable psychological status. Without clear systems or processes it was acknowledged that making these decisions could be difficult.

*There should not be any ambiguity about which patients it is that we should think about… If one is to be able to identify the right patient, one must really know what you are looking for, otherwise it is quite difficult*. (focus group 1)

Consequently, physicians often needed to adapt the criteria for each unique clinical setting. This could also include using their own intuition or involving the wider care team in discussions about whether certain patients needed or were ready for SIC.

## Discussion

The aim of the study was to explore physicians’ perceptions and experiences of the implementation of the SICP. A variety of features across different systems and levels were perceived to influence the implementation. Physicians described individual, team, organizational, and processual aspects as instrumental for SICP implementation. The physicians were impacted by their experiences, preparedness, and views about the SICP, as well as interactions and negotiations between and within cultural and system-level contexts. These professional, implementation, and system features and processes were described as dynamic and multifaceted. These perceptions provide guidance for sustainable integration and future SICP implementations. The findings will be discussed through the lens of the Consolidated Framework for Implementation Research (CFIR). The CFIR can be used to assess contextual factors that may impact interventions and can be applied by implementation practitioners (i.e. to guide implementation), as well by implementation scientists (i.e. to study implementation) [14]. The CFIR domains: innovation, outer setting, inner setting, individuals, and implementation process highlight aspects that are central for the success of an implementation and the factors that may hinder it [15].

According to CFIR, the roles and characteristics of individuals are recognized to influence implementations, including professional attributes related to needs, capabilities, and motivation [14, 15]. Individual and professional views and capacity for having SIC were described in this study as a process of uncovering and building awareness of strengths and weaknesses which was linked to communication confidence and competence. This study also found that shared views and beliefs were held among the physicians that this kind of communication was valuable and should be a part of their daily work. Paladino et al. [16] reiterated that clinicians’ receptiveness to SICP and adoption of SIC was important, but could be influenced by clinical or societal culture. For instance, having SIC can interfere with beliefs that the physician’s professional role is to “treat”, not to talk about values and goals [16]. Physicians’ attitudes and their own capabilities in SIC have been described as both an enabler and a barrier [17]. However, altering mindsets and creating culture shifts regarding attitudes and norms around SIC can occur alongside the implementation process [18]. This supports the idea that implementations such as the SICP can contribute towards culture change in the palliative care specialty [19] and foster a shared moral responsibility for care [20].

The need for a team-based approach was highlighted in the present study. The physicians noted that it was necessary to engage the wider healthcare team in patient identification for SIC. The importance and meaning of interprofessional collaboration in palliative care and SIC has also been emphasized in other areas [21], including training and supporting all team members in the SICP components to facilitate a shared vision within the team, formation of clear professional roles, and shared accountability [22]. A well-functioning team-based SICP implementation can add perspective and value from nonphysician team members and distribute workload and responsibilities throughout the team [23]. Subsequently, to build a strong team culture to facilitate implementation of SICP, other professions within the multidisciplinary healthcare team should be involved in all parts of the implementation process. This is supported by Meyers et al.’s [24] assertion that developing the social aspect is a process where formal and informal relationships influence implementation success. The findings from the present study reveal the need for formal structures and forums to support the team to plan, discuss, debrief, and reflect on the experience and progress of the SICP implementation. Adjustments were made by the team to fit SIC into everyday work processes, such as changing criteria for specific clinical settings or in relation to the SICG structure, e.g., using all of the SICG, or parts of the SICG. However, the SICP structure was largely adhered to, which is an indicator of process quality (cf. 15).

The need to adapt identification methods was recognized for specific settings (e.g., units or departments), as well as for different patient groups (e.g., based on diagnosis) and patient conditions (e.g., illness progression, mental and physical health status). In past research, Swedish physicians and nurses described ethical and existential obstacles in identifying patients for SIC with regard to timing, relationships and attitudes [25]. A recent scoping review showed that patient identification for SIC can be undertaken in various ways and by different clinicians, including physicians, nurses, and allied health [26]. Patients were primarily identified using the surprise question or clinical/diagnostic triggers, but a combination of manual or automated identification methods could be applied [26]. Development of routines for patient identification should be undertaken on-site by clinicians, with consideration given to practice culture and patient population [27]. This means that identification strategies should be evaluated and refined during the implementation process [27] to identify the right patient at the right time instead of adopting a one-size fits-all approach [25]. Processes such as these can be impacted by structural characteristics and clinical culture aspects within the inner setting of an organization [15]. The findings highlight the importance of flexibility in workflow processes, such as patient identification, and indicate that organizational preparedness and cooperation is a necessity when implementing the SICP.

Effective education is another critical step in implementation [24]. In our findings, the SIC training was perceived to facilitate access to knowledge and skills development through discovery and acknowledgement of communication strengths and weaknesses. However, physicians suggested that there was a need for some kind of continuous training or development through peer support. This was particularly true regarding the topic of prognosis, which was described by the participating physicians as being difficult to address. Daubman et al., [28] describe that physician level of comfort or discomfort discussing prognosis is known to vary and can be reliant on individual experience and cultural aspects within the profession and workplace. Geerse et al. [29] found that clinicians did not address all elements of the SICG in every conversation, with the prognosis element discussed least despite the majority of the patients in the study indicating that they wanted prognostic information. In this way, it is necessary to consider the surrounding support systems for implementations [15], such as the wider hospital system and community. The external sociocultural context may have limited the implementation due to societal discomfort with the topic of death and dying. Not wanting to cause harm by speaking about prognosis, provoking anxiety or taking away hope emerged as key aspects. To mitigate this risk, physicians emphasized the importance of fostering awareness around shared values and developing a collective sense of responsibility to support patients and families through SIC.

The perceived design, quality, advantage, and adaptability must be considered in relation to implementation success [14, 15]. Physicians preferred SIC to the traditional way of communicating and it was valued as a way of humanizing care. The SICG was viewed as a resource for learning how to lead SIC. However, physicians identified the need for modification of materials and to be able make adaptations to suit different patient populations, communication styles, and identification methods. The findings illuminated how physicians adhered to and adapted the SICP and SICG. Modification of materials in the SICP is encouraged [30] and clinicians should adapt SIC according to their own style and the needs of individual patients [31]. Nevertheless, it is essential to distinguish between the core and adaptable components of an innovation [15]. The original SICG defined several key elements: illness understanding, decision-making and information preferences, prognostic disclosure, patient goals and fears, views on acceptable function and trade-offs, and family involvement [3]. While adaptations have been made to the SICG in real-world implementations, a recent systematic review found that the general structure and questions were relevant for most clinical contexts [32]. Future research should explore which aspects of the SICP are essential or unchangeable to assess and secure fidelity in SICP implementations.

### Strengths and limitations

The methods used in this study require consideration. Regarding trustworthiness [33], the credibility of the study was enhanced by purposive recruitment of participants based on their involvement in the SICP implementation. This ensured that participants were well-positioned to provide rich data that aligned with the overall aim of the study. In addition, quotations were provided to support the results. A transparent description of all steps taken in conducting this study strengthens dependability. Given that the SICP was implemented at two hospitals in Sweden, the transferability of these findings may be limited and therefore warrants further exploration in other settings (cf. 33).

The focus group discussion method can be viewed both as a strength and a limitation. Participants may not have felt able to express themselves freely in front of their colleagues, especially when discussing the team culture, due to fear of judgement, disagreement, or reprisal (cf. 8). However, it is important to note that the data expressed both positive and negative perceptions of the SICP indicating that participants felt comfortable to talk about their experiences. Despite some participants voicing ‘negative’ experiences, they were also able to identify areas for improvement. The number of participants in the focus group discussions ranged from two to five per group which may have impacted discussion dynamics; however, the discussions lasted between 37 and 61 min which indicates that the smaller groups were also able to engage in rich discussions. The participants within each focus group came from the same workplace which could also have impacted the content and direction of the discussions. By conducting four focus groups with participants from different clinical areas it was possible to explore a range of experiences in relation to SICP implementation. Lastly, it is not possible to know the extent to which physicians practiced SIC before or after the training. However, as this study was part of a novel implementation of the SICP participants were unlikely to have encountered or used SIC previously. Future studies could explore the impact of time spent practicing SIC in relation to uptake of SICP implementation.

## Conclusions

This study identified key aspects at individual, team, and organizational levels which were perceived to influence the implementation of SICP. Undertaking SIC can be challenging, it is therefore essential to provide physicians with training and ongoing support, as well as the ability to adapt or modify parts of the SIC or SICP to their own personal or professional preferences. Further research is needed to explore how healthcare professionals’ attitudes may impact the SICP implementation process and how it may affect its sustainability over time. This study reports on an initial effort to outline meaningful constructs and aspects for implementation of SICP from the point of view of physicians. The findings highlight both the strength of the implementation strategies to drive change, as well as the considerable effort that remains for ongoing implementation success.

### Electronic supplementary material

Below is the link to the electronic supplementary material.

Supplementary Material 1

Supplementary Material 2

## Data Availability

The data that support the findings of this study are available from the corresponding author upon reasonable request.

## Abbreviations

CFIR: Consolidated Framework for Implementation Research
SIC: Serious Illness Conversations
SICP: Serious Illness Care Program
SICG: Serious Illness Conversation Guide
