# Supplementary material for: Physicians’ perceptions of the implementation of the serious illness care program: a qualitative study
Source: BMC Health Serv Res. 2023 Dec 12;23:1401. doi: 10.1186/s12913-023-10419-5 (PMC10717999; doi:10.1186/s12913-023-10419-5)
Supplement: Supplementary file 1 — Supplementary Material 1 [file 12913_2023_10419_MOESM1_ESM.docx]

**Supplementary File A**

**Focus group discussion guide**

Discussion topics regarding thoughts/perceptions/experiences connected to:

- the serious illness care program in practice
- identification of patients for serious illness conversations
- the serious illness conversation training
- communication with seriously ill patients
- using the serious illness conversation guide
