# Supplementary material for: Physicians’ perceptions of the implementation of the serious illness care program: a qualitative study
Source: BMC Health Serv Res. 2023 Dec 12;23:1401. doi: 10.1186/s12913-023-10419-5 (PMC10717999; doi:10.1186/s12913-023-10419-5)
Supplement: Supplementary file 2 — Supplementary Material 2 [file 12913_2023_10419_MOESM2_ESM.docx]

**Supplementary File B**

**Table s. 1. Consolidated criteria for reporting qualitative studies (COREQ): 32-item checklist**

| **Item** | **CORE-Q checklist questions** | **Page** |
| --- | --- | --- |
| **Domain 1: Research team and reﬂexivity** | | |
| *Personal Characteristics* | | |
| 1. Interviewer/facilitator | Which author/s conducted the interview or focus group? | **6, 22** |
| 2. Credentials | What were the researcher’s credentials? | **Title page** |
| 3. Occupation | What was their occupation at the time of the study? | **6** |
| 4. Gender | Was the researcher male or female? | **6** |
| 5. Experience and training | What experience or training did the researcher have? | **6** |
| *Relationship with participants* | | |
| 6. Relationship established | Was a relationship established prior to study commencement? | **6** |
| 7. Participant knowledge of the interviewer | What did the participants know about the researcher? | **6** |
| 8. Interviewer characteristics | What characteristics were reported about the interviewer/ facilitator? | **6** |
| **Domain 2: Study Design** | | |
| 9. Methodological orientation and Theory | What methodological orientation was stated to underpin the study? | **4** |
| *Participant selection* | | |
| 10. Sampling | How were participants selected? | **5** |
| 11. Method of approach | How were participants approached? | **5** |
| 12. Sample size | How many participants were in the study? | **5** |
| 13. Non-participation | How many people refused to participate or dropped out? Reasons? | **5** |
| *Setting* | | |
| 14. Setting of data collection | Where was the data collected? | **6** |
| 15. Presence of non-participants | Was anyone else present besides the participants and researchers? | **6** |
| 16. Description of sample | What are the important characteristics of the sample? | **5-6** |
| *Data collection* | | |
| 17. Interview guide | Were questions, prompts, guides provided by the authors? Was it pilot tested? | **6** |
| 18. Repeat interviews | Were repeat interviews carried out? If yes, how many? | **N/A** |
| 19. Audio/visual recording | Did the research use audio or visual recording to collect the data? | **6** |
| 20. Field notes | Were ﬁeld notes made during and/or after the interview or focus group? | **N/A** |
| 21. Duration | What was the duration of the interviews or focus group? | **6** |
| 22. Data saturation | Was data saturation discussed? | **19-20** |
| 23. Transcripts returned | Were transcripts returned to participants for comment and/or correction? | **N/A** |
| **Domain 3: analysis and ﬁndings** | | |
| *Data analysis* | | |
| 24. Number of data coders | How many data coders coded the data? | **22** |
| 25. Description of the coding tree | Did authors provide a description of the coding tree? | **6-7** |
| 26. Derivation of themes | Were themes identiﬁed in advance or derived from the data? | **6-7** |
| 27. Software | What software, if applicable, was used to manage the data? | **N/A** |
| 28. Participant checking | Did participants provide feedback on the ﬁndings? | **N/A** |
| *Reporting* | | |
| 29. Quotations presented | Were participant quotations presented to illustrate the themes/ ﬁndings? Was each quotation identiﬁed? | **8-14** |
| 30. Data and ﬁndings consistent | Was there consistency between the data presented and the ﬁndings? | **7-15** |
| 31. Clarity of major themes | Were major themes clearly presented in the ﬁndings? | **7-15** |
| 32. Clarity of minor themes | Is there a description of diverse cases or discussion of minor themes? | **7-15** |

Developed from: Tong, A., Sainsbury, P., & Craig, J. (2007). Consolidated criteria for reporting qualitative research (COREQ): a 32-item checklist for interviews and focus groups. *International Journal for Quality in Health Care, 19*(6), 349 – 357.
